# Influence of a Socially Assistive Robot on Physical Activity, Social Play Behavior, and Toy-Use Behaviors of Children in a Free Play Environment: A Within-Subjects Study

**DOI:** 10.3389/frobt.2021.768642

**Published:** 2021-11-22

**Authors:** Joseline Raja Vora, Ameer Helmi, Christine Zhan, Eliora Olivares, Tina Vu, Marie Wilkey, Samantha Noregaard, Naomi T. Fitter, Samuel W. Logan

**Affiliations:** ^1^ Social Mobility Lab, College of Public Health and Human Sciences, Oregon State University, Corvallis, OR, United States; ^2^ Collaborative Robotics and Intelligent Systems (CoRIS) Institute, Oregon State University, Corvallis, OR, United States

**Keywords:** assistive robotics, robot-mediated play, physical activity, play behavior, toy use behavior

## Abstract

**Background:** Play is critical for children’s physical, cognitive, and social development. Technology-based toys like robots are especially of interest to children. This pilot study explores the affordances of the play area provided by developmentally appropriate toys and a mobile socially assistive robot (SAR). The objective of this study is to assess the role of the SAR on physical activity, play behavior, and toy-use behavior of children during free play.

**Methods:** Six children (5 females, M_age_ = 3.6 ± 1.9 years) participated in the majority of our pilot study’s seven 30-minute-long weekly play sessions (4 baseline and 3 intervention). During baseline sessions, the SAR was powered off. During intervention sessions, the SAR was teleoperated to move in the play area and offered rewards of lights, sounds, and bubbles to children. Thirty-minute videos of the play sessions were annotated using a momentary time sampling observation system. Mean percentage of time spent in behaviors of interest in baseline and intervention sessions were calculated. Paired-Wilcoxon signed rank tests were conducted to assess differences between baseline and intervention sessions.

**Results:** There was a significant increase in children’s standing (∼15%; *Z* = −2.09; *p* = 0.037) and a tendency for less time sitting (∼19%; *Z* = −1.89; *p* = 0.059) in the intervention phase as compared to the baseline phase. There was also a significant decrease (∼4.5%, *Z* = −2.70; *p* = 0.007) in peer interaction play and a tendency for greater (∼4.5%, *Z* = −1.89; *p* = 0.059) interaction with adults in the intervention phase as compared to the baseline phase. There was a significant increase in children’s interaction with the robot (∼11.5%, *Z* = −2.52; *p* = 0.012) in the intervention phase as compared to the baseline phase.

**Conclusion:** These results may indicate that a mobile SAR provides affordances through rewards that elicit children’s interaction with the SAR and more time standing in free play. This pilot study lays a foundation for exploring the role of SARs in inclusive play environments for children with and without mobility disabilities in real-world settings like day-care centers and preschools.

## Background

The Office of Disease Prevention and Health Promotion reports that physical activity for children improves bone health, cardiorespiratory and muscular fitness, cognitive skills, concentration in tasks, and body fat content (Kohrt et al., 2004; Warburton, 2006; Kohl and Cook, 2013). Yet, about half of preschool-aged children do not engage in the recommended amount of physical activity throughout the day (Tucker, 2008). The United States Department of Health and Human Services recommends that preschool-aged children be active all day (Physical Activities Guidelines for Americans, 2nd Edition, 2018). Preschool-aged children spend a considerable amount of time in free play. Free play is an important context to observe children’s behavior because it provides autonomy for children to engage with peers, toys, and the environment. Free play is also an under-studied context for the use of robots with young children. However, it is concerning that only a portion of free play time is spent in moderate to vigorous physical activity (Verstraete et al., 2006). Our work considers the use of robots as a potential means to encourage children to engage in moderate to vigorous physical activity during free play. One of the goals of this study was to incorporate the use of a SAR in a free play setting. In a real-world context such as a playground, it is likely that children of varying ages will be in the same environment and engage in free play together. Play with children of different ages may also occur with siblings. Therefore, we included children with a wide variety of ages to provide a real-world context for use of the SAR. In addition, we were interested in if the SAR had broad appeal to children of different ages; our pilot study was an appropriate first step to determine this feasibility.

The affordance of a play area, defined as the environment it provides to a child, plays an important role in the child’s active engagement in play (Herrington and Brussoni, 2015). Modifying the play area with children’s evolving interests to stimulate various senses and promote social interactions are strategies to keep children excited about play time (Shackell, 2008). In the past decades different kinds of robotic toys, including socially assistive robots (SAR) have made their way into play spaces (Lund, 1999; Michaud, Duquette and Nadeau, 2003; Bian et al., 2020). In a closely related work by our study team using a complementary set of annotated data compared to the current manuscript, we observed greater engagement of children with a SAR that was mobile in the play area, and provided visual, tactile, and auditory rewards to children, as compared to SAR stationary conditions (Vinoo et al., 2021).

This paper presents a study that introduces an infant-sized mobile SAR in a free play environment. The objectives of our study are to enhance the affordances of the play area by providing developmentally appropriate toys and a SAR. We aim to assess the influence of a mobile SAR with rewards of lights, sounds, and bubbles on physical activity, play behavior, and toy-use behavior of children in a free play environment. The study consists of a baseline phase and intervention phase. In the baseline phase, the SAR is an inactive (i.e., immobile and powered off) part of the play area. In the intervention phase, the SAR is mobile and adds to the affordances of the play space by offering rewards (lights, sounds, and bubbles) to children. In this paper, we describe related work (*Related Work Section*) that informed our aims, outline the methods (*Methods Section*), report our main findings (*Results Section*), discuss the results (*Discussion Section*), note limitations and future work (*Limitations and Future Work Section*), and summarize the conclusions from our study (*Conclusion Section*).

### Related Work

Key past work discussed in this section has enabled us to develop our study objectives and design our study. Broadly, we are interested in exploring the affordances provided by the SAR in children’s play behavior. The United Nations recognizes the importance of play and recreational activities and regards them as a child’s basic right (United Nations Convention on the Rights of the Child, 1990). In describing play, Pellegrini and Smith (1998) summarize play as an enjoyable activity that children engage in without a specific purpose.

#### Play Behavior and Child Development

Play has a vital role in a child’s life and development (Vygotsky, 1967; Ahmad et al., 2016). Play helps a child grow physically, emotionally, and cognitively (Thomas and Harding, 2011), and serves as a conduit to explore the environment and interact with peers, adults, and objects such as toys (Logan et al., 2015, 2016).

Play has been defined in different ways over the past few centuries and is difficult to define in a single sentence (Miller, 1973). Aristotle associated play with freedom and self-sufficiency (Besio et al., 2017). Play has been described as an activity that although is not considered serious, completely engrosses the individual (Huizinga, 2014). According to Piaget (1952), play develops from a stage of sensorimotor activity to a more advanced symbolic or imaginative play. Further, Piaget also stated that assimilation and accommodation are factors that determine a child’s adaptation to its environment (Besio et al., 2017; Piaget, 1952). Graham and Burghardt (2010) took a broader lens in describing play, stating that five criteria need to be met for a behavior to be categorized as play. These criteria for play behavior require the activity to be: 1) not completely functional in its context; 2) voluntary, spontaneous, enjoyable, and rewarding to the individual; 3) different from more serious behaviors in terms of its form and timing, meaning that play is usually exaggerated and that children engage in play much earlier in life than they engage in other serious behaviors; 4) usually repeated; and 5) often engaged in when an individual is not in severe stress.

Exposure to various kinds of play involving multiple motor skills early in life predicts a child’s ability to excel in those skills and learn new motor skills later in life. For example, a child who has learned an overhand throw can build on this skill to learn various sport like tennis, badminton and volleyball later on (Bunker, 1991). Play has also been shown to improve social interaction and reduce disruptive behavior in schools, especially among children with intellectual disabilities. Engaging in play that starts at the skill level of the child and gradually challenges the child to enhance their play skills helps the child connect better with their peers (O'Connor and Stagnitti, 2011). Finally, play, especially physical activity play, contributes to the cognitive development of the child as well. Participating in physical activity play wherein children engage in moderate to vigorous physical activity during play leads to a feeling of arousal or vigilance. An appropriate amount of arousal due to physical activity enhances mental performance (Pellegrini and Smith, 1998; Shepard, 1983). Interventions like the Sitting Together and Reaching to Play (START-Play) that incorporated cognitive constructs and motor challenges into play are effective in improving cognitive outcomes in infants as young as 7 months of age. Infants with severe motor delays who underwent the START-Play intervention showed advanced problem-solving skills and cognitive ability compared to their counterparts receiving the usual early intervention care (Harbourne et al., 2021). These studies exemplify the significance of play in the physical, social, and cognitive development of children.

Play behavior advances as a child grows and develops, as listed in Table 1. For example, play during infancy is associated with exploration of the environment (Rusher et al., 1995), engagement with adults, and interaction with objects, including toys (Bradley, 1985). Infancy (three to 18 months) is typically associated with solitary play that mostly occurs independently of nearby people. Although it is most dominant in early months of life, children also engage in solitary play later in childhood (Anderson-McNamee and Bailey, 2010). Toddlerhood (18–24 months) is typically associated with parallel play that occurs when children start playing in proximity with other children, but without actively interacting with them (Howes and Matheson, 1992). Early childhood (three to 4 years) is typically associated with peer interaction that may be associative or social play. Associative play is when a child becomes interested in a peer’s toys and interacts with them with the intention of playing with their toys. Social play may include when children cooperate with each other and share toys to interact (Anderson-McNamee and Bailey, 2010). Apart from toys and peers, children also engage with adults in the play area. Adults interact actively or passively with children in the play area, especially in early stages of development (Parten, 1932). Our study seeks to explore changes in play behavior in children at various developmental stages as a result of the affordances provided by the SAR in the play area.

**TABLE 1:** Predominant play behavior based on age.

Age range	Predominant play behavior
3–18 months	Solitary Play
18–24 months	Parallel Play
3–4 years	Peer interaction play, Adult interaction play

#### Affordances and Child Development

Affordances within a play area provide children with opportunities to explore the world. These may include substances, objects, and persons in the environment (Gibson, 1977). In a child’s play area, affordances may be provided by toys, peers, and adults who facilitate play. The availability of developmentally appropriate toys enhances the quality of play (Trawick-Smith et al., 2015). The number and variety of toys available during play contribute towards improving the child’s development, including cognitive (Bradley, 1985) and motor (Abbott and Bartlett, 2001) skills. Children up to the age of eight prefer exploratory, building, pretend play, physical activity and recreational, learning, sensory, and technological toys (Richards et al., 2020). Among technology-based toys, one that is of broad interest to toddlers and older children alike are robotic toys. Although these toys appeal to children, little work to date has explored the influence of mobile robots on children’s free play.

#### Socially Assistive Robots

SARs have been used extensively to teach children cognitive, social, and motor skills (Marino et al., 2020). Fitter et al. (2019) reported that infants as young as 6 months of age could imitate knee extension ball-kick behavior demonstrated by the Aldebaran NAO SAR. While some infants in this study imitated the SAR without any rewards, others showed greater kicking acceleration when rewarded by lights or sounds generated by the robot. Guneysu and Arnrich (2017) demonstrated feasibility of using the humanoid NAO robot in one-on-one exercise instruction and imitation by children. In another study, the NAO and the Wonder Workshop Dash (a small, wheeled toy robot) were part of a robot-assisted learning environment in a child rehabilitation setting (Kokkoni et al., 2020). Two infants and a toddler supported by a body weight support system exhibited complex motor tasks like climbing when following the robots. Children also aided the robots to complete motor tasks like going up an inclined surface, tasks that these toys were unable to complete without assistance.

Among the few studies that explored the impact of robots in a social setting was the work by Kozima and Nakagawa (2007) who introduced an interactive Keepon robot to a group of preschoolers. They report initiation of interactions between the robot and children, and among children themselves in the presence of the robot. The lifelike properties or animism of robots plays an important role in captivating the interest of children (Melson et al., 2009; Beran et al., 2011). Researchers and practitioners capitalize on children’s interest in robots to teach skills for which traditional teaching/therapy techniques may not be as effective. For example, there are multiple interventions for children with autism that employ robots to teach psychomotor skills such as movement in the four main directions (Moorthy and Pugazhenthi, 2017), coach children on the recognition of social and gestural cues, and enhance communication skills (Cabibihan et al., 2013; So et al., 2018). Robot imitation has also been used extensively, especially with children with autism, to teach skills through play and physical activity (Robins et al., 2008; Conti et al., 2015). For example, in one past study, two children with autism engaged in a game of imitation and turn-taking with the Kaspar humanoid robot (Robins et al., 2008). The goal of this robot study was to teach children with autism to engage in interactive play while learning skills in turn-taking by imitating the robot’s posture change over time. Robot imitation has also inculcated the participating children with social skills.

SARs have also been used extensively to promote efficient learning in children of various ages, skill levels, and abilities. For example, a study by Hsiao et al. (2015) reported that children between the ages of two and three have greater literacy in a language when they practiced reading with a “learning buddy” that was an SAR as compared to a tablet PC. The bidirectional modality of learning with the SAR was highly effective in keeping children engaged to learn more content. In another study involving the NAO robot as a learning peer among primary school children, researchers reported that with personalization, the SAR helped children learn a novel task while keeping them interested in the task through the entire study (Baxter et al., 2017). These studies provide evidence for SARs to be seamlessly incorporated with existing technology to motivate children in active learning.

Many of these interventions use SARs to engage participants in social interactions (Feil-Seifer and Mataric, 2005). There is, however, a dearth of research using mobile SARs, particularly those with a child-sized form factor and a base motion speed capable of matching the pace of children during moderate to vigorous physical play activity. Our SAR, which includes a TurtleBot 2 base, possesses this combination of compelling size and suitable motion speed for active play.

## Methods

The study involved seven weekly free play sessions with a within-subjects group design. During the first four sessions (baseline phase), the SAR was powered off. In the last three sessions (intervention phase), the SAR was teleoperated to move in the play area and offered rewards of lights, sounds, and bubbles to children.

### Participants

Six children between the ages of one and seven (Range = 1.6–6.7 years; M = 3.6; SD = 1.9; five females; all Caucasian), who attended two or more play sessions during both the baseline and intervention phases were included for analyses.

### Procedure

#### IRB and Informed Consent

Approval for all study procedures was obtained from the Oregon State University Institutional Review Board. Written informed consent from parents was obtained prior to the start of the study.

### Play Area

**FIGURE 1:**
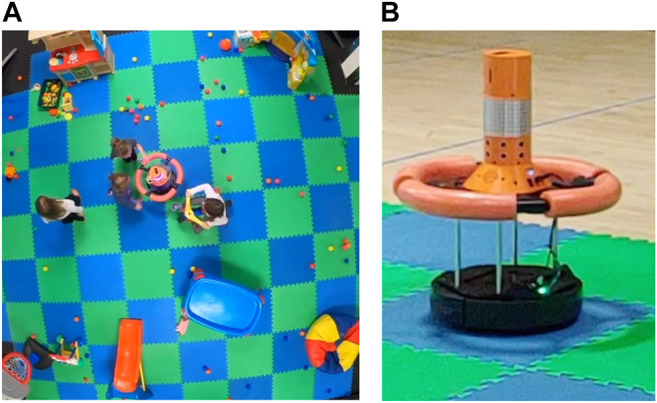
**A** Overhead view of the play area with developmentally appropriate toys and the SAR; **B** SAR in the play area.

The play area (approximately 440 sq. ft or 41Melson et al., 2009m^2^) was lined with alternately colored blue and green foam squares (each 2′ X 2’ or 0.6 × 0.6 m), and children were instructed to remain in the play area for the entire session. At the start of each play session, developmentally appropriate toys were set up in the same location, as shown in Figure 1. Toys for the age range included physical activity and recreational toys, sensory toys, learning toys, pretend play toys, and the SAR.

### Play Session Description

There was a total of seven weekly sessions with four baseline sessions (weeks one to four) and three intervention phases (weeks five to seven). A fourth planned weekly session for the intervention phase was cancelled due to the COVID-19 pandemic. Each weekly session was approximately 30 min long wherein children engaged in free play. In this study, free play is defined as play behavior that is controlled by the child, with minimal involvement of adults (O’Brien and Smith, 2002). Parents and research team members intervened minimally during play time. All adults stayed in the periphery of the play area. Adults were instructed to intervene in children’s play only when the interaction was initiated by a child or when the intervention was necessary (e.g., when requested to intervene due to a potential safety issue, to facilitate sharing of toys between children).

The SAR used in the study was an infant-sized mobile robot which is capable of providing configurable rewards of lights, sounds, and bubbles. The TurtleBot 2 base of the SAR houses a Raspberry Pi 3 B+, which controls the release of the rewards, and a 12 V power supply. The reward stack was 3D-printed with orange and clear polylactic acid (PLA). The SAR could provide six animations for the light rewards in different colors and frequencies. It was programmed to produce 200 different types of sounds (e.g., musical notes, animal noises) that were inspired by infant toys. Finally, the SAR had a bubble-producing module that consisted of a motor and a radial fan to make and dispense bubbles.

During the baseline phase the SAR was powered off, and during the intervention phase, a research team member used a teleoperation interface to maneuver the SAR to approach each child in the play area at varying intervals and activated the rewards of lights, sounds, or bubbles. We randomized the order in which children were approached each session using a random number generator. Every child received all three rewards during every play session in the intervention phase.

### Data Collection and Video Coding

Overhead GoPro cameras were used to record the 30-min play sessions, and these videos were used for data analyses.

### Measurement

As summarized in Table 2, physical activity, play behavior, and toy-use behavior variables were annotated based on a predefined codebook, and the child and robot positions were tracked using computer vision.

**TABLE 2:** Behavior assessments with categories.

Behavior	Categories
Physical Activity Type	Standing
	Climbing
	Lying
	Jumping
	Sitting/Squatting
	Sliding Down
	Bending
	Riding
	Walking
	Crawling
	Lifting
	Throwing/Catching
	Kneeling
	Running
	Pulling/Pushing
	Walking on Knees
Play Behavior	Unoccupied play-Child not engaging in any play behavior
	Solitary play-Child playing independently without interaction with anyone
	Parallel play-Child playing within three ft of another child without deliberate interaction with the peer
	Peer interaction play-Child engaging in direct verbal or physical interaction with peer
	Adult interaction play-Child engaging in direct verbal or physical interaction with adult
Toy-use Behavior	Physical activity recreation toys-Mini basketball unit, slides, walkers, balls and trike
	Sensory toys-Sensory table and bean bag chair
	Learning toys-Play unit and activity tables
	Pretend play toys-Play kitchen, play food, play mobile phone, hand puppets and shopping cart
	SAR

#### Physical Activity

Physical activity behaviors were adapted from a direct observation system called the Observational System for Recording Physical Activity in Children: Elementary School (OCRAC-E; McIver et al., 2016) to add more behaviors like catching/throwing, riding, and walking on knees based on observed behaviors during playgroup sessions (Table 2). The OSRAC observation system is used commonly to record children’s physical activity behaviors (Pate et al., 2013; Logan et al., 2015).

#### Play Behavior

Play behaviors were adapted from the Parten’s Stages of Play (Parten, 1932), and Peer Play Scale (Howes and Matheson, 1992). The adaptations from both of these scales were made to include behaviors of interest for the current study. Similar coding systems have been used to assess play behavior of children at various stages of development (Pellegrini and Perlmutter, 1989; Howes and Matheson, 1992; Logan et al., 2015). Play behaviors were categorized as unoccupied play, solitary play, parallel play, peer interaction play, and adult interaction play (Table 2).

#### Toy-Use Behavior

Toy-use behaviors were annotated based on the type of toy children were interacting with. Developmentally appropriate toys for the age range included physical activity and recreational toys, sensory toys, learning toys, pretend play toys, and the SAR (Table 2). The categorization of these toys was done as per the standard ‘Age Determination Guidelines’ (Richards et al., 2020).

#### Child and SAR Positioning

Positional data for the child and SAR were extracted using the OpenCV multi-object tracking function (Rosebrock, 2017) in a custom Python script. This region-of-interest tracker is commonly used in several different contexts such as traffic surveillance, surgery, and medical imaging since it allows for position monitoring for entities of interest (Doukas and Maglogiannis, 2007). To use the script, a research assistant selected bounding boxes for the SAR and each child in the play area. If a child or the SAR left the play area, the research assistant would re-select this target of interest when that child or robot re-entered the frame. At a rate of 25 frames per second, position data was automatically recorded at each timestep based on the position of each bounding box’s center.

### Data Analysis

The videos were annotated using a momentary time sampling observation system (Brown et al., 2006; Logan et al., 2015). This technique involves breaking down the 30 min of video into 10-s consecutive intervals, observing the child behavior for the first 2 seconds of each interval, and recording the observed behaviors during the remaining 8 seconds of each interval. The protocol used in this study was adapted from previous studies where the first 5 seconds of 15-s intervals (Logan et al., 2015) or 25-s intervals (Brown et al., 2006) were annotated for child behaviors. Shorter epochs of 10 s were used for recording behaviors in the present work based on accelerometer-based cut-point estimations for moderate to vigorous physical activity of toddlers (Trost et al., 2012). Six observation intervals were annotated for each minute, resulting in 180 observations per child for every session. This yielded in a total of 5,400 observation intervals across the study.

Two trained coders annotated all the video recordings for behaviors. One coder annotated the physical activity behaviors, and the other coder annotated play behavior and toy-use behavior. An inter-rater reliability of at least 85% agreement was established between an additional expert coder and the two trained coders for 10% of the video recordings. Agreement of 85% or higher is considered acceptable in observational studies of children (Logan et al., 2015). Percent agreement was calculated as the following:

$$Percent\;Agreement=\;\left(\frac{a}{a+\;b}\right)\times \;100$$

where a = # of agreements, and b = # of disagreements between coders.

For physical activity, play behavior, and toy-use behaviors, the percentage of total intervals when the child was in the field of view is reported. For child and robot positioning, the percentage of total frames when the child was within three feet of the robot in the field of view is reported. For each child, mean percentage of time spent in each behavior in each individual phase is calculated as follows:

$$Mean\;\%\;of\;Time\;in\;Behavior=\;\left(\frac{c}{d}\right)\times 100$$

where c = # of observed intervals for the behaviour, and d = Total # of intervals.

For the computer vision-generated data the distance between the SAR and each child was calculated for every timestep. Then, the percentage of frames where the child was within three feet of the SAR was calculated to determine time spent by the child in parallel or more complex play behaviors within close proximity of the robot (Howes and Matheson, 1992). For each child, mean percentage of time spent that the child was within three feet of the SAR is calculated as follows:

$$Mean\;\%\;of\;Time\;frames\;when\;child\;was\;within\;3\;ft\;of\;SAR=\;\left(\frac{e}{f}\right)\times 100$$

where e = # of time frames when the distance between child and SAR is <3ft, and f = Total # of time frames.

### Statistical Analyses

A within-subjects group design was used to analyze the data. Due to the non-parametric nature of the data, paired Wilcoxon signed rank tests were conducted using the SPSS statistical software (version 25).

## Results

In this section, we provide the breakdown of coding results for each behavioral assessment of interest. In each of the figures below, we first report behaviors for individual play sessions during the baseline and intervention phases. Then, we report behaviors combined across baseline and intervention phases. All of the SAR technology worked correctly for all sessions except the bubble-blowing attachment, which had reduced functioning during Session 6, the second session of the intervention phase.

### Physical Activity Type

**FIGURE 2:**
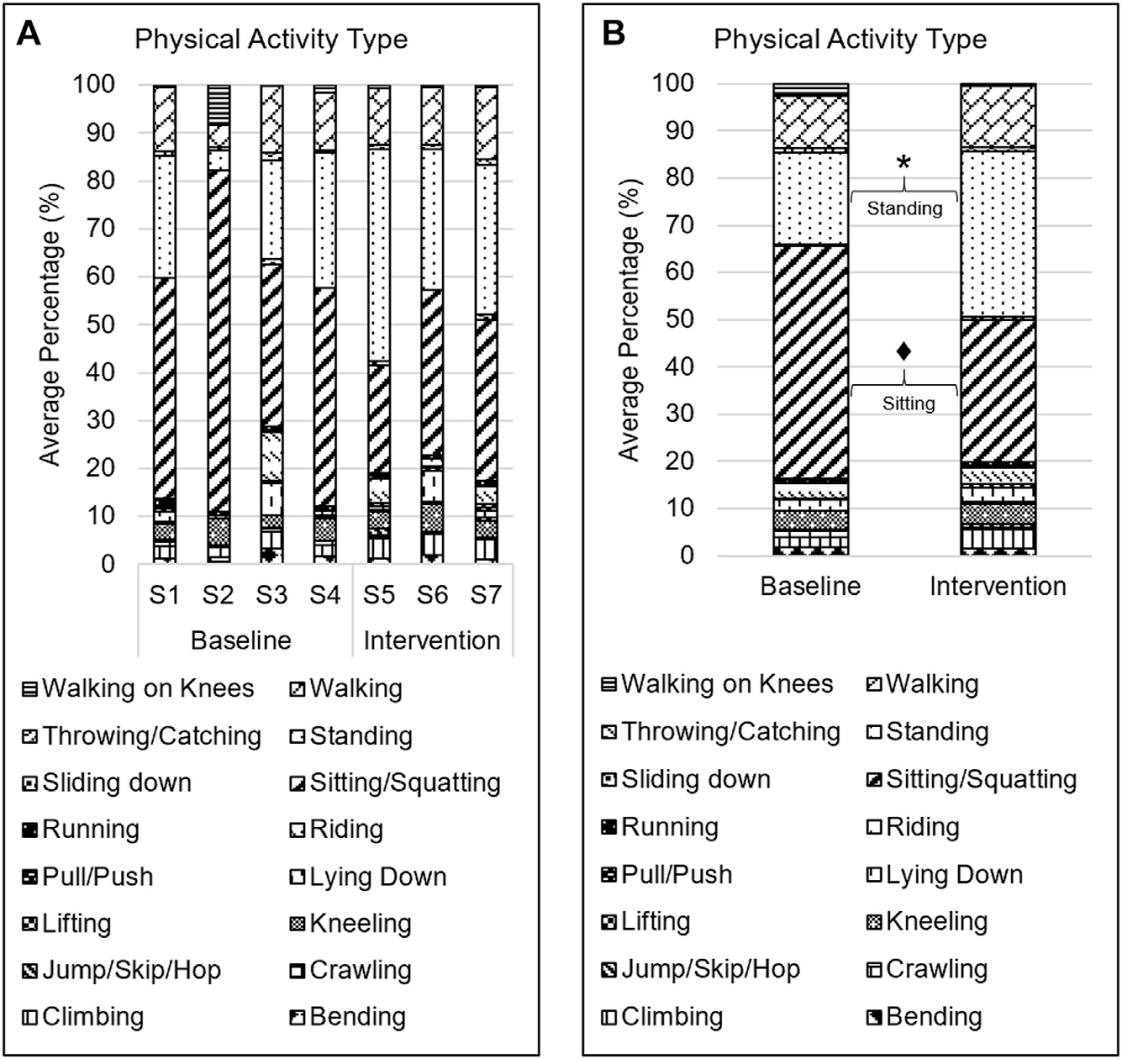
Physical activity type. **A**. Average percentage of intervals spent in each type of physical activity across sessions. S# on the x-axis represents the session number. **B**. Average percentage of intervals spent in each type of physical activity during baseline and intervention phases. * → *p* < 0.05; ♦ → *p* < 0.06.

Children engaged in all types of physical activity during the play sessions (Figures 2A,B). Much of the play time was spent in three types of physical activity including sitting/squatting (∼40%) standing (∼27%), and walking (∼12%). The other physical activity types accounted for a combined total of ∼21% of time intervals. Time spent in standing was significantly greater (∼15%; *Z* = −2.09; *p* = 0.037) in the intervention phase as compared with baseline phase (Figure 2B). Conversely, time spent in sitting tended to be lesser (∼19%; *Z* = −1.89; *p* = 0.059) in the intervention phase as compared with baseline phase. There were no significant differences in time spent in other physical activity types between baseline and intervention phases.

### Play Behavior

**FIGURE 3:**
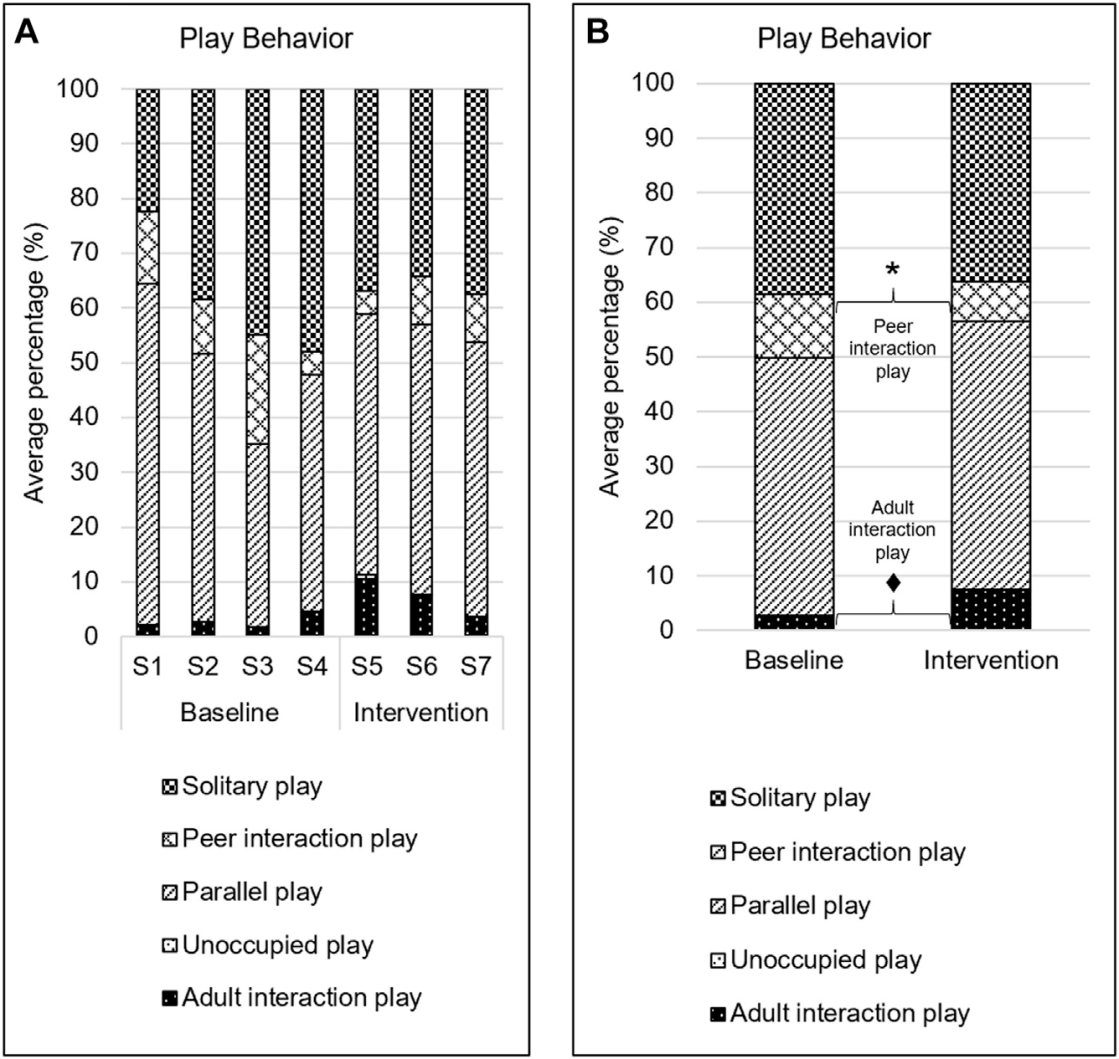
Play behavior. **A**. Average percentage of intervals spent in each type of play behavior. S# on the *x*-axis represents the session number. **B**. Average percentage of intervals spent in each type of play behavior during baseline and intervention phases. * → *p* < 0.05; ♦ → *p* < 0.06.

Children spent majority of the play time engaged in parallel play (∼48%) followed by solitary play (∼37%; Figures 3A,B). There was a significant decrease (∼4.5%, *Z* = −2.70; *p* = 0.007) in peer interaction play in the intervention phase as compared to the baseline phase (Figure 3C). There was a tendency for greater (∼4.5%, *Z* = −1.89; *p* = 0.059) interaction with adults in the intervention phase as compared to the baseline phase.

### Toy-Use Behavior

**FIGURE 4:**
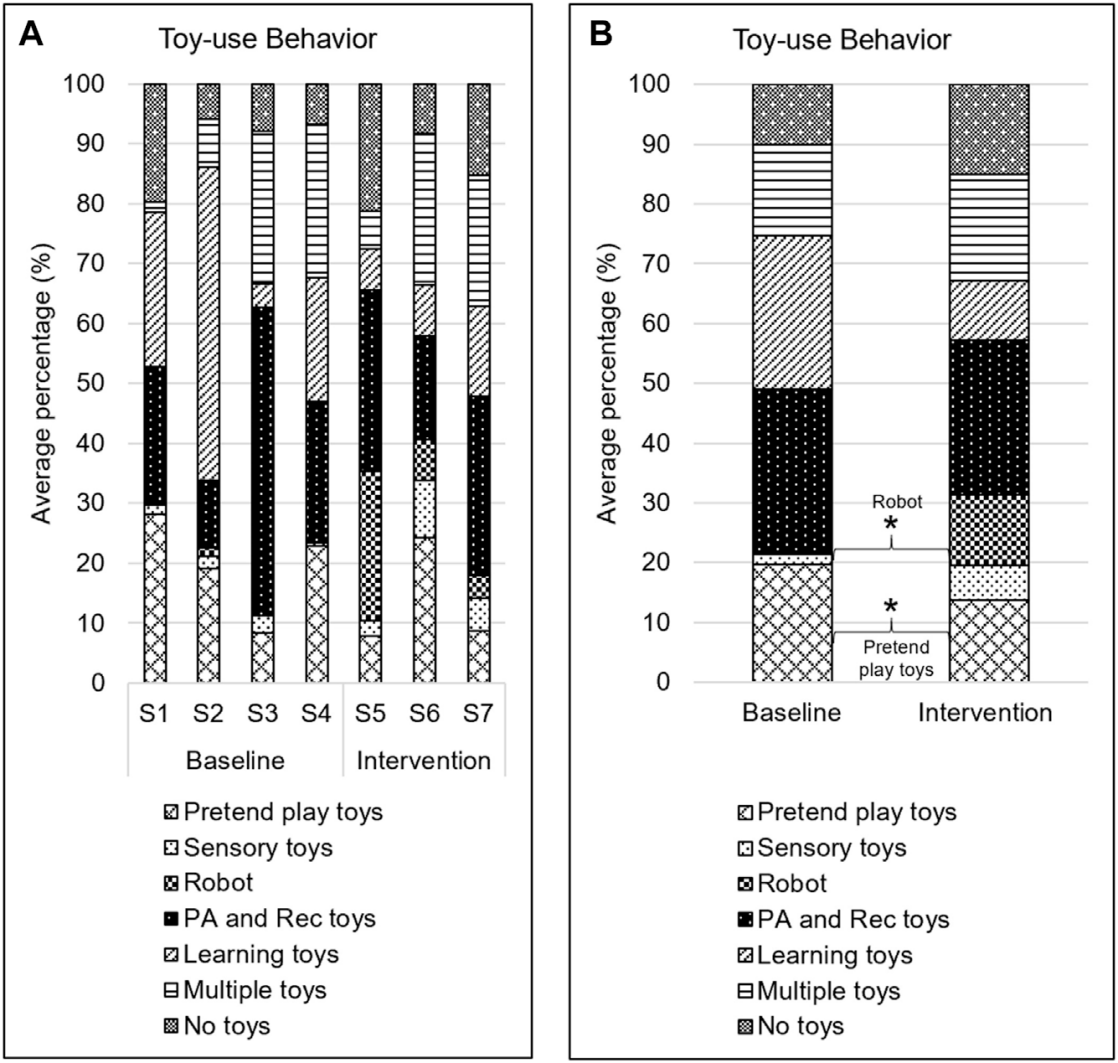
Toy-use behavior. **A**. Average percentage of intervals spent interacting with each type of toys. S# on the *x*-axis represents the session number. **B**. Average percentage of intervals spent playing with each type of toy during baseline and intervention phases. * → *p* < 0.05.

Children played with a variety of toys including physical activity and recreation toys (∼26.5% of time intervals), learning toys (∼18% of time intervals), pretend play toys (∼17% of time intervals), the SAR (∼5% of time intervals), and sensory toys (∼4% of time intervals) throughout the study. They also played with multiple toys (∼16.5% of time intervals) at a time and engaged in play with no toys (∼12.5% of time intervals). There was a significant increase (∼11.5%, *Z* = −2.52; *p* = 0.012) in interaction with the robot in the intervention phase compared to baseline phase. These interactions included touching, following, looking at, pushing/pulling, or going towards the robot or its rewards (Vinoo et al., 2021). There was also a significant decrease (∼6%, *Z* = −2.40; *p* = 0.017) in play with pretend-play toys in the intervention phase as compared to baseline phase (Figure 4).

### Child and SAR Positioning

**FIGURE 5:**
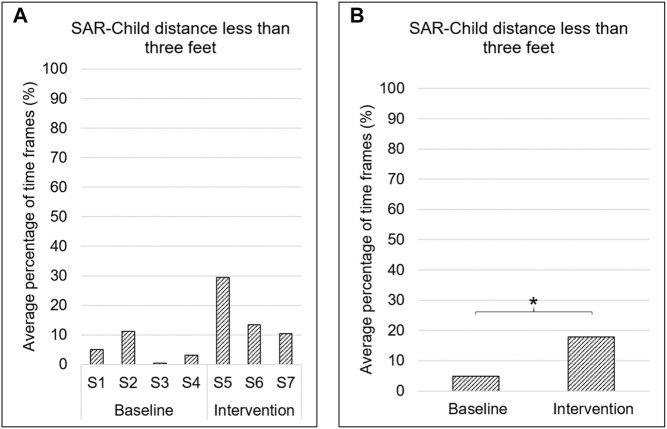
SAR-child positioning. **A**. Average percentage of time frames when children were within three feet of the robot. S# on the *x*-axis represents the session number. **B**. Average percentage of time frames when children were within three feet of the robot during baseline and intervention phases. * → *p* < 0.05.

While interacting with the robot, children spent ∼10.5% of time within three feet of the robot throughout the study. Children spent significantly greater time (∼12.9%; *p* = 0.02) within three feet of the robot in the intervention phase as compared to the baseline phase (Figure 5B).

## Discussion

This is the first study to introduce a mobile SAR in a free play environment to assess children’s physical activity, play behavior, toy-use behavior, and proximity to the robot. Enhanced child behaviors during the intervention phase when the robot was active suggest potential effects of the affordance provided by the mobile SAR in its design and the rewards of lights, sounds, and bubbles.

Children spent more time standing and had a tendency to sit less in the intervention phase compared to the baseline phase. A parallel segment of this study with the SAR reports that children look at and touch the robot more when it is mobile (Vinoo et al., 2021). It is possible that children were captivated by the novelty of the SAR and its rewards, hence spent more time standing and engaging with it as it approached them and their peers (Parten, 1932). Children spent less time standing and more time sitting in the second and third intervention sessions (Session six and seven of the current study, respectively), as compared to the session when the SAR was first activated. This observation implies that the novelty of the robot dwindles over time. The fading of ‘novelty effect’ is common especially in studies involving children (Leite et al., 2014; Serholt and Barendregt, 2016). Leite et al. (2014), for example, conducted a study with an iCat social robot that played chess with children over 5 weeks. The research team reported that children noticed the robot less during the later weeks as compared to the earlier weeks. Similar to our study, this work attributed the waning interest of children in the robot to the novelty effect. Strategies to address the declining novelty effect include using SARs with life-like properties, staggered introduction of SAR novel behaviors, and making the SAR more adaptive to child behaviors. Gradually introducing children to different robot behaviors, rather than all at once is also likely to keep them more engaged with the robot. Kokkoni et al. (2020) noted that children are more likely to look at the robot if it exhibits unexpected behaviors. Finally, reciprocal behavior from the SAR in the form of verbal or non-verbal social interactions may also be key to keep children interested in the SAR (Castellano et al., 2008).

In the intervention phase, peer interaction play decreased with a corresponding tendency of increased adult interaction as compared to baseline phase. Over the study period, as children familiarized themselves with the play environment and adults (including research staff and parents), they initiated more conversations, pretend play, and other play behaviors with adults. For example, a child brought play food on a plate to one of the research team members, while another child initiated a game of catch with other research team members. Additionally, in the intervention phase, children interacted more with the robot teleoperator to initiate more robot rewards, especially bubble rewards. Although research team members were in the outer perimeter of the play area, this finding suggests that adults are an important affordance of a child’s play area (Sando and Sandseter, 2020). In their study with a socially expressive Pleo dinosaur robot and children with ASD, Kim et al. (2013) report that children initiated spontaneous conversations with an unfamiliar adult predominantly about the robot. Our study varied slightly from the work of Kim et al., in that our participants had seen and interacted with adults for 4 weeks before the intervention phase when the SAR was powered on. However, like in the past study with Pleo, our participants had questions about, and expressed excitement and interest towards, the robot, especially in the first intervention session (session 5). Solitary play, parallel play, and intervals when children were not playing did not vary significantly between the baseline and intervention phases.

## Limitations and Future Work

A limitation of our study is the small sample size (*n* = 6) with limited diversity (five females, all Caucasian, all typically developing). Also, we had limited intervention sessions owing to the COVID-19 pandemic and could not incorporate a withdrawal phase following the intervention with the robot turned off again, similar to the baseline phase. Our study design consisted of non-randomized baseline and intervention phases, leading to possible ordering effects in our observations. Another limitation of our study was the inability to control for the adult interaction with participants in the study. It is possible that even with the provided instructions, adults may have initiated extra interactions, and we did not control for the source of initiated interactions in our analyses. Finally, we had minor technical difficulties with the SAR in Session 6, leading to reduced functioning of the bubble-blowing attachment during the latter part of that session.

This pilot study contributes towards the limited literature on affordances provided by a mobile SAR in a free play environment. Future work needs to expand on the current findings by increasing the sample size and purposefully recruiting children for a more diverse and inclusive playgroup. These findings also demonstrate the feasibility of using mobile SARs in social settings like classrooms, daycare centers, playgrounds, and parks for purposes apart from education and skill-development. The next steps will be to study the role of mobile SARs on physical activity, play behavior, and toy-use behaviors in these natural settings with a broader user base, including children with disabilities.

## Conclusion

The current study incorporated an infant-sized mobile SAR in the play space to assess its influence on children’s physical activity, play behavior and toy-use behavior. Results of the current study suggest that the SAR is capable of engaging children’s attention through increased proximity and play with the SAR during the intervention. Interest in, and engagement with the SAR when it moves and provides rewards demonstrates the affordance it provides to engage children in the play area. Greater interaction and closer proximity to the robot may also be attributed to the novelty of a mobile SAR with rewards. Another novel finding of our study was that our SAR encouraged children to stand more than sit during play. These findings pave a path for employing SARs in combination with assistive mobility technologies like the body weight support system, walkers, motorized wheelchairs and orthoses to augment engagement and exploration of the environment by children with mobility disabilities. Furthermore, toy companies can focus on developing SARs that offer a wide array of developmentally appropriate rewards, to engage children with and without disabilities in various kinds of moderate to vigorous physical activity.

## Data Availability

Based on the terms of our IRB protocol, datasets collected from this study are not publicly available. These datasets are only available to those individuals added to the IRB protocol.

## Abbreviations

SAR, Socially Assistive Robot.

## References

[B1] Abbott A. L. Bartlett D. J. (2001). Infant Motor Development and Equipment Use in the home. Child. Care Health Develop. 27 (3), 295–306. 10.1046/j.1365-2214.2001.00186.x 11350456

[B2] Ahmad S. Abid Hussain C. Batool A. Sittar K. Malik M. (2016). Play and Cognitive Development: Formal Operational Perspective of Piaget's Theory. J. Educ. Pract. 7 (28), 72–79.

[B3] Anderson-McNamee J. K. Bailey S. J. (2010). The Importance of Play in Early Childhood Development, 4. Bozeman: Montana State University Extention, 1–4.

[B4] Baxter P. Ashurst E. Read R. Kennedy J. Belpaeme T. (2017). Robot Education Peers in a Situated Primary School Study: Personalisation Promotes Child Learning. PloS one 12 (5), e0178126. 10.1371/journal.pone.0178126 28542648 PMC5441605

[B5] Beran T. N. Ramirez-Serrano A. Roman K. Fior M. Nugent S. (2011). Understanding How Children Understand Robots: Perceived Animism in Child–Robot Interaction. Int. J. Human Comp. Stud. 69 (7-8), 539–550. 10.1016/j.ijhcs.2011.04.003

[B6] Besio S. Bulgarelli D. Stancheva-Popkostadinova V. (2017). Play Development in Children with Disabilties. Warsaw: De Gruyter Open Poland.

[B7] Bian Y. Wang X. Han D. Sun J. (2020). “Designed Interactive Toys for Children with Cerebral Palsy,” in Proceedings of the Fourteenth International Conference on Tangible, Embedded, and Embodied Interaction, Sydney, February 9 - 12, 2020 (Sydney NSW Australia: ACM), 473–478. 10.1145/3374920.3374975

[B8] Bradley R. H. (1985). Social-Cognitive Development and Toys. Top. Early Child. Spec. Educ. 5 (3), 11–29. 10.1177/027112148500500303

[B9] Brown W. H. Pfeiffer K. A. McIver K. L. Dowda M. Almeida J. M. C. A. Pate R. R. (2006). Assessing Preschool Children's Physical Activity. Res. Q. Exerc. Sport 77 (2), 167–176. 10.1080/02701367.2006.10599351 16898273

[B10] Bunker L. K. (1991). The Role of Play and Motor Skill Development in Building Children's Self-Confidence and Self-Esteem. Elem. Sch. J. 91 (5), 467–471. 10.1086/461669

[B11] Cabibihan J.-J. Javed H. Ang M. Aljunied S. M. (2013). Why Robots? A Survey on the Roles and Benefits of Social Robots in the Therapy of Children with Autism. Int. J. Soc. Robot. 5 (4), 593–618. 10.1007/s12369-013-0202-2

[B12] Castellano G. Aylett R. Dautenhahn K. Paiva A. McOwan P. W. Ho S. (2008). “Long-term Affect Sensitive and Socially Interactive Companions,” in Proceedings of the 4th International Workshop on Human-Computer Conversation, Bellagio, Italy, October 6, 2008, 1–5.

[B13] Conti D. Di Nuovo S. Buono S. Trubia G. Di Nuovo A. (2015). “Use of Robotics to Stimulate Imitation in Children with Autism Spectrum Disorder: A Pilot Study in a Clinical Setting,” in 2015 24th IEEE international symposium on robot and human interactive communication (RO-MAN), Kobe, Japan, August 31-September 4, 2015 (IEEE), 1–6. 10.1109/roman.2015.7333589

[B14] Doukas C. Maglogiannis I. (2007). Region of Interest Coding Techniques for Medical Image Compression. IEEE Eng. Med. Biol. Mag. 26 (5), 29–35. 10.1109/EMB.2007.901793 17941320

[B16] Feil-Seifer D. Mataric M. J. (2005). “Socially Assistive Robotics,” in 9th International Conference on Rehabilitation Robotics, 2005. ICORR 2005, Chicago, June 28-July 1, 2005 (Chicago, IL, USA: IEEE), 465–468. 10.1109/ICORR.2005.1501143

[B17] Fitter N. T. Funke R. Pulido J. C. Eisenman L. E. Deng W. Rosales M. R. (2019). Socially Assistive Infant-Robot Interaction: Using Robots to Encourage Infant Leg-Motion Training. IEEE Robot. Automat. Mag. 26 (2), 12–23. 10.1109/MRA.2019.2905644

[B18] Gibson G. D. (1977). Himba Epochs. Hist. Afr. 4 (no. 2), 67–121. 10.2307/3171580

[B70] Graham K. L. Burghardt G. M. (2010). Current perspectives on the biological study of play: signs of progress. Quat. Rev. Biol. 85 (4), 393–418. 10.1086/656903 21243962

[B19] Guneysu A. Arnrich B. (2017). “Socially Assistive Child-Robot Interaction in Physical Exercise Coaching,” in 2017 26th IEEE International Symposium on Robot and Human Interactive Communication (RO-MAN), Lisbon, Portugal, August 28-September 1, 2017 (Lisbon: IEEE), 670–675. 10.1109/ROMAN.2017.8172375

[B20] Harbourne R. T. Dusing S. C. Lobo M. A. McCoy S. W. Hsu L. Y. Willett S. (2021). START-play Physical Therapy Intervention Impacts Motor and Cognitive Outcomes in Infants with Neuromotor Disorders: A Multisite Randomized Clinical Trial. Phys. Ther. 101 (2), pzaa232. 10.1093/ptj/pzaa232 33382406 PMC7910024

[B21] Herrington S. Brussoni M. (2015). Beyond Physical Activity: The Importance of Play and Nature-Based Play Spaces for Children's Health and Development. Curr. Obes. Rep. 4 (4), 477–483. 10.1007/s13679-015-0179-2 26399254

[B22] Howes C. Matheson C. C. (1992). Sequences in the Development of Competent Play with Peers: Social and Social Pretend Play. Develop. Psychol. 28 (5), 961–974. 10.1037/0012-1649.28.5.961

[B23] Hsiao H.-S. Chang C.-S. Lin C.-Y. Hsu H.-L. (2015). "iRobiQ": the Influence of Bidirectional Interaction on Kindergarteners' reading Motivation, Literacy, and Behavior. Interactive Learn. Environ. 23 (3), 269–292. 10.1080/10494820.2012.745435

[B24] Huizinga J. (2014). Homo Ludens Ils 86. New York: Routledge.

[B25] Kim E. S. Berkovits L. D. Bernier E. P. Leyzberg D. Shic F. Paul R. (2013). Social Robots as Embedded Reinforcers of Social Behavior in Children with Autism. J. Autism Dev. Disord. 43 (5), 1038–1049. 10.1007/s10803-012-1645-2 23111617

[B26] Kohl H. W. Cook H. D. (2013). Educating the Student Body: Taking Physical Activity and Physical Education to School. Washington, D.C.: The National Academies Press. 24851299

[B27] Kohrt W. M. Bloomfield S. A. Little K. D. Nelson M. E. Yingling V. R. (2004). Physical Activity and Bone Health. Med. Sci. Sports Exerc. 36 (11), 1985–1996. 10.1249/01.MSS.0000142662.21767.58 15514517

[B28] Kokkoni E. Mavroudi E. Zehfroosh A. Galloway J. C. Vidal R. Heinz J. (2020). GEARing Smart Environments for Pediatric Motor Rehabilitation. J. Neuroeng. Rehabil. 17 (1). 10.1186/s12984-020-0647-0 PMC7011606 32041623

[B29] Kozima H. Nakagawa C. (2007). “A Robot in a Playroom with Preschool Children: Longitudinal Field Practice,” in RO-MAN 2007 - The 16th IEEE International Symposium on Robot and Human Interactive Communication, Jeju, South Korea, August 26-29, 2007 (Jeju, South Korea: IEEE), 1058–1059. 10.1109/ROMAN.2007.4415238

[B30] Leite I. Castellano G. Pereira A. Martinho C. Paiva A. (2014). Empathic Robots for Long-Term Interaction. Int. J. Soc. Robot. 6 (3), 329–341. 10.1007/s12369-014-0227-1

[B31] Logan S. W. Ross S. M. Schreiber M. A. Feldner H. A. Lobo M. A. Catena M. A. (2016). Why We Move: Social Mobility Behaviors of Non-disabled and Disabled Children across Childcare Contexts. Front. Public Health 4. 10.3389/fpubh.2016.00204 PMC5030269 27709110

[B32] Logan S. W. Schreiber M. Lobo M. Pritchard B. George L. Galloway J. C. (2015). Real-World Performance. Pediatr. Phys. Ther. 27 (4), 433–441. 10.1097/PEP.0000000000000181 26397093

[B34] Lund H. H. (1999). “AI in Children’s Play with LEGO Robots,” in Proceedings of AAAI 1999 Spring Symposium Series, Orlando, July 18-22, 1999.

[B35] Marino F. Chilà P. Sfrazzetto S. T. Carrozza C. Crimi I. Failla C. (2020). Outcomes of a Robot-Assisted Social-Emotional Understanding Intervention for Young Children with Autism Spectrum Disorders. J. Autism Dev. Disord. 50 (6), 1973–1987. 10.1007/s10803-019-03953-x 30852783

[B36] McIver K. L. Brown W. H. Pfeiffer K. A. Dowda M. Pate R. R. (2016). Development and Testing of the Observational System for Recording Physical Activity in Children: Elementary School. Res. Q. Exerc. Sport 87 (1), 101–109. 10.1080/02701367.2015.1125994 26889587 PMC4762057

[B37] Melson G. F. Peter H. Beck A. Friedman B. Roberts T. Garrett E. (2009). Children’s Behavior toward and Understanding of Robotic and Living Dogs. J. Appl. Develop. Psychol. 11, 92–102. 10.1016/j.appdev.2008.10.011

[B38] Michaud F. Duquette A. Nadeau I. (2003). “Characteristics of Mobile Robotic Toys for Children with Pervasive Developmental Disorders,” in SMC’03 Conference Proceedings. 2003 IEEE International Conference on Systems, Man and Cybernetics. Conference Theme - System Security and Assurance (Cat. No.03CH37483), Washington, October 8, 2003 (Washington, DC, USA: IEEE), 2938–2943. 10.1109/ICSMC.2003.1244338

[B69] Miller S. (1973). Ends, Means, and Galumphing: Some Leitmotifs of Play 1. Amer. Anthropologist 75 (1), 87–98.

[B39] Moorthy R. S. Pugazhenthi S. (2017). Teaching Psychomotor Skills to Autistic Children by Employing a Robotic Training Kit: A Pilot Study. Int. J. Soc. Robot. 9 (1), 97–108. 10.1007/s12369-016-0375-6

[B41] O'Brien J. Smith J. (2002). Childhood Transformed? Risk Perceptions and the Decline of Free Play. Br. J. Occup. Ther. 65 (3), 123–128. 10.1177/030802260206500304

[B42] O’Connor C. Stagnitti K. (2011). Play, Behaviour, Language and Social Skills: The Comparison of a Play and a Non-play Intervention within a Specialist School Setting. Res. Develop. Disabilities 32 (3), 1205–1211. 10.1016/j.ridd.2010.12.037 21282038

[B43] Parten M. B. (1932). Social Participation Among Pre-school Children. J. Abnormal Soc. Psychol. 27 (3), 243–269. 10.1037/h0074524

[B44] Pate R. R. Dowda M. Brown W. H. Mitchell J. Addy C. (2013). Physical Activity in Preschool Children with the Transition to Outdoors. J. Phys. Activity Health 10 (2), 170–175. 10.1123/jpah.10.2.170 22820709

[B45] Pellegrini A. D. Perlmutter J. C. (1989). Classroom Contextual Effects on Children's Play. Develop. Psychol. 25 (2), 289–296. 10.1037/0012-1649.25.2.289

[B46] Pellegrini A. D. Smith P. K. (1998). Physical Activity Play: The Nature and Function of a Neglected Aspect of Play. Child. Develop. 69 (3), 577–598. 10.1111/j.1467-8624.1998.tb06226.x 9680672

[B47] Piaget J. (1952). Play, Dreams and Imitation in Childhood. Oxon: Routledge.

[B48] Piercy K. L. Troiano R. P. Ballard R. M. Carlson S. A. Fulton J. E. Galuska D. A. (2018). The Physical Activity Guidelines for Americans. Jama 320 (19), 2020–2028. 10.1001/jama.2018.14854 30418471 PMC9582631

[B49] Richards M. N. Putnick D. L. Suwalsky J. Bornstein M. H. (2020). Age Determination Guidelines: Relating Consumer Product Characteristics to the Skills, Play Behaviors, and Interests of Children. Consumer Product Safety Commission.

[B50] Robins B. Ferrari E. Dautenhahn K. (2008). “Developing Scenarios for Robot Assisted Play,” in RO-MAN 2008-The 17th IEEE International Symposium on Robot and Human Interactive Communication, Munich, Germany, August 1-3, 2008 (IEEE), 180–186. 10.1109/roman.2008.4600663

[B51] Rosebrock A. (2017). Object Detection with Deep Learning and OpenCV. Open CV resources.

[B52] Rusher A. S. Cross D. R. Ware A. M. (1995). Infant and Toddler Play: Assessment of Exploratory Style and Development Level. Early Child. Res. Q. 10 (3), 297–315. 10.1016/0885-2006(95)90009-8

[B53] Sando O. J. Sandseter E. B. H. (2020). Affordances for Physical Activity and Well-Being in the ECEC Outdoor Environment. J. Environ. Psychol. 69 (June), 101430. 10.1016/j.jenvp.2020.101430

[B54] Serholt S. Barendregt W. (2016). “Robots Tutoring Children,” in Proceedings of the 9th Nordic Conference on Human-Computer Interaction, Gothenburg, October 23-27, 2016 (Gothenburg Sweden: ACM), 1–10. 10.1145/2971485.2971536

[B55] Shackell A. (2008). Design for Play: A Guide to Creating Successful Play Spaces. Nottingham: Department for Children, Schools and Families. Available at: http://publications.everychildmatters.gov.uk/eOrderingDownload/Design%20for%20Play.pdf .

[B56] Shephard R. J. (1983). Physical Activity and the Healthy Mind. Can. Med. Assoc. J. 128 (5), 525–530. 6337692 PMC1874986

[B57] So W.-C. Wong M. K.-Y. Lam W.-Y. Chui A. T.-F. Lee T.-L. Ng H.-M. (2018). Using a Social Robot to Teach Gestural Recognition and Production in Children with Autism Spectrum Disorders. Disabil. Rehabil. Assistive Techn. 13 (6), 527–539. 10.1080/17483107.2017.1344886 28673117

[B59] Thomas F. Harding S. (2011). “The Role of Play: Play Outdoors as the Medium and Mechanism for Well-Being, Learning and Development,” in Outdoor Provision in the Early Years, by Jan White (London, United Kingdom: SAGE Publications Ltd), 12–22. 10.4135/9781446289099.n2

[B60] Trawick-Smith J. Wolff J. Koschel M. Vallarelli J. (2015). Effects of Toys on the Play Quality of Preschool Children: Influence of Gender, Ethnicity, and Socioeconomic Status. Early Child. Educ. J. 43 (4), 249–256. 10.1007/s10643-014-0644-7

[B61] Trost S. G. Fees B. S. Haar S. J. Murray A. D. Crowe L. K. (2012). Identification and Validity of Accelerometer Cut-Points for Toddlers. Obesity 20 (11), 2317–2319. 10.1038/oby.2011.364 22173573

[B62] Tucker P. (2008). The Physical Activity Levels of Preschool-Aged Children: A Systematic Review. Early Child. Res. Q. 23 (4), 547–558. 10.1016/j.ecresq.2008.08.005

[B63] Unicef (1990). The United Nations Convention on the Rights of the Child. Available at: http://www.unicef.org.uk/Documents/Publication-pdfs, UNCRC_PRESS200910web.pdf (Accessed August 28, 2021).

[B64] Verstraete S. J. M. Cardon G. M. De Clercq D. L. R. De Bourdeaudhuij I. M. M. (2006). Increasing Children's Physical Activity Levels during Recess Periods in Elementary Schools: the Effects of Providing Game Equipment. Eur. J. Public Health 16 (4), 415–419. 10.1093/eurpub/ckl008 16431866

[B65] Vinoo A. Case L. Zott G. R. Raja Vora J. Helmi A. Logan S. W. (2021). “Design of an Assistive Robot for Infant Mobility Interventions,” in 2021 30th IEEE International Conference on Robot & Human Interactive Communication (RO-MAN), Vancouver, BC, Canada, August 8-12, 2021 (IEEE), 604–611. 10.1109/ro-man50785.2021.9515415

[B66] Vora J. R. Helmi A. Zhan C. Olivares E. Vu T. Wilkey M. (2021). Influence of a Socially Assistive Robot on Physical Activity, Play Behavior, and Toy-Use Behaviors of Children in a Free Play Environment: A Within-Subjects Study. Research square. 10.3389/frobt.2021.768642 PMC8645936 34881295

[B67] Vygotsky L. S. (1967). Play and its Role in the Mental Development of the Child. Soviet Psychol. 5 (3), 6–18. 10.2753/RPO1061-040505036

[B68] Warburton D. E. R. (2006). Health Benefits of Physical Activity: The Evidence. Can. Med. Assoc. J. 174 (6), 801–809. 10.1503/cmaj.051351 16534088 PMC1402378

